# Quantifying muscle contraction with a conductive electroactive polymer sensor: introduction to a novel surface mechanomyography device

**DOI:** 10.1080/23335432.2024.2319068

**Published:** 2024-02-28

**Authors:** Donna Moxley Scarborough, Shannon E. Linderman, Ryan Aspenleiter, Eric M. Berkson

**Affiliations:** a Department of Orthopaedic Surgery, Massachusetts General Hospital, Boston, Massachusetts, USA; b Research & Development, FIGUR8, Inc, Boston, USA

**Keywords:** Muscle contraction measurement, wearable, dynamometer, electromyography

## Abstract

Clinicians seek an accurate method to assess muscle contractility during activities to better guide treatment. We investigated application of a conductive electroactive polymer sensor as a novel wearable surface mechanomyography (sMMG) sensor for quantifying muscle contractility. The radial displacement of a muscle during a contraction is detected by the physically stretched dielectric elastomer component of the sMMG sensor which quantifies the changes in capacitance. The duration of muscle activation times for quadriceps, hamstrings, and gastrocnemius muscles demonstrated strong correlation between sMMG and EMG during a parallel squat activity and isometric contractions. A moderate to strong correlation was demonstrated between the sMMG isometric muscle activation times and force output times from a dynamometer. The potential wearable application of an electroactive polymer sensor to measure muscle contraction time is supported.

## Introduction

Measures quantifying the timing of a muscle’s ability to contract (i.e. contractility) are desirable for improving motor performance, injury recovery and injury avoidance (Nazmi et al. 2016; Macgregor et al. 2018; Pereira et al. 2019). Currently, clinicians progress patients through treatment stages using timelines primarily derived from muscle imaging studies, not functional information about a muscle’s ability to activate or sustain a contraction (Sherry et al. 2015). Wearable sensor technology offers various methods of capturing different highly valued metrics throughout a muscle contraction including during functional activities. Surface electromyography (sEMG) is the clinical standard for assessing the electrical signal identifying the timing of muscle activation during static and dynamic activities (Basmajian and De Luca 1985; Nazmi et al. 2016). However, there are several data collection and analysis challenges, such as the potential for signal interference and the need for complex signal processing, that limit the utility of EMG for wide-spread application during clinically relevant exercise and movement assessments (Madeleine et al. 2001; Ibitoye et al. 2014; Pilkar et al. 2020). Mechanomyography (MMG), a methodology for assessing the mechanical activity of muscles during contraction, has recently gained greater attention as an adjunct to sEMG (Oster and Jaffe 1980; Madeleine et al. 2001; Macgregor et al. 2018; Esposito et al. 2018). While these MMG systems purport insight into physical properties, such as the timing of a muscle contraction, many are limited in practicality for clinical application (Ibitoye et al. 2014; Esposito et al. 2018). An electroactive polymer sensor with a flexible construction offers potential as a novel application for capturing the physical properties of a muscle contraction. Dielectric elastomer sensors are a type of electroactive polymer sensor which can measure strain through the detection of change in capacitance as the sensor is stretched. The flexible design of this sensor conforms to the body which facilitates easy and secure attachment for capturing muscle contraction data, further supporting its investigation as a new method of surface mechanomyography for future clinical application. This content validation study presents the foundational step for determining the potential utilization of an electroactive sensor for clinical application. We investigated the feasibility and validation of the electroactive polymer sensor to capture the duration of muscle activation time during a set of isometric muscle contractions and during a commonly used movement screening activity, the parallel squat. Comparison of the timing of muscle contraction recorded by the electroactive polymer sensors with simultaneously collected force dynamometer data during isometric resisted muscle tests was also included in this study.

Electroactive polymer sensor designs offer the ability to easily conform to human body segments (Wang et al. 2016). These dielectric sensors are relatively inexpensive to fabricate, light, and report stability in working cycles (Wang et al. 2016). These sensors have been applied in micro-robotics, orthotics, and prosthetics, and were more recently explored for measuring human kinematics (Bar-Cohen 2002; Esposito et al. 2018). Current manufacturing allows electroactive polymer sensors to transmit data via Bluetooth, easing their application as wearable devices (Wang et al. 2016).

We introduce the application of an electroactive polymer sensor for characterizing muscle contractility and will refer to the output and application of this sensor as sMMG (surface mechanomyography) in this paper. The fine measurement of area displacement of the dielectric elastomer film with 0.01 mm resolution allows for the recording of superficial measurement of muscle bulk change. In this initial content validity study, we hypothesized that the duration of the muscle contraction time provided by the sMMG will correlate with 1) the duration of muscle contraction captured by sEMG during maximum volitional isometric contractions and during the parallel squat activity and 2) the duration of contraction captured by force output from a dynamometer during maximum volitional isometric contractions for three lower extremity muscles (quadriceps, hamstrings and gastrocnemius).

## Methods

### Participants

Fifteen healthy, active individuals (mean age = 33.1 ± 9.71 y, height: 177.43 ± 7.90 cm, weight: 77.93 ± 14.37 kg) participated in this cross-sectional study. Nine males and six females provided informed consent to participate in this institutional review board approved study (2017P000975). All subjects had no history of lower extremity or back surgery, fracture or neurological disorder that influences movement patterns. Participants were required to be free of self-reported injury 3-months prior to enrollment.

### Surface mechanomyography sensor hardware and software

**Figure 1.**
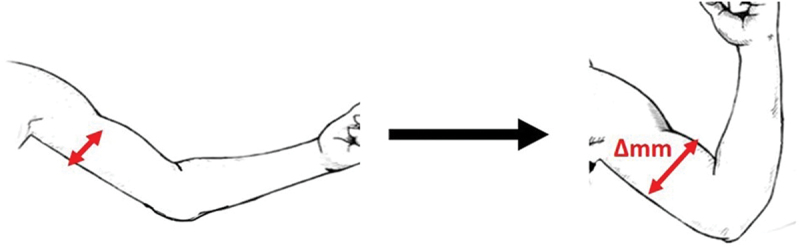
Illustration of electroactive capacitive sensor (surface mechanomyography) application across the bulk of a muscle and lengthens as the muscle contracts (depicted in red).

The sMMG sensor is a first-generation dielectric elastomer from a vendor’s research evaluation kit (FIGUR8, Inc, Boston, MA). The electroactive polymer-based capacitive displacement sensor reports 0.01 mm resolution with a 1000 mm dynamic range (Linderman 2023). The sensor stiffness is reported at 0.065 N/mm and is calibrated to read 0 mm when at 105 mm (resting length), which gives a 105–205 mm measurement range (Linderman 2023). The muscle bulk deformation beneath the sensor was calculated by the impedance change of the dielectric electroactive film within the sensor. When the sensor is strained (stretched) it thins and expands in area, increasing its capacitance (Figure 1). This displacement provided by the sensor represents the change in muscle radial displacement, which translates to information about muscle contraction function, such as timing of muscle activation, amplitude and contractility when viewed in a time series. The sMMG sensor transmits data via Bluetooth Low Energy (BLE) wireless data streaming at 50 Hz. The data was collected using a mobile app on an iPhone 8.

### Surface electromyography hardware and software

The sEMG Trigno Avanti Electromyography System (Figure 3) was collected at 1925 Hz and streamed via BLE to a laptop (Delsys, Natick, MA). The wireless sEMG sensors (27 × 37 × 13 mm, 14 g) were placed directly on the skin superficial to the muscle of interest. The sEMG sensor records the monopolar electrical signal from the active motor unit action potentials during a muscle contraction (Basmajian and De Luca 1985). The Delsys EMGworks® Acquisition Software was used to record data, and the Delsys File Utility was used to convert data for analysis.

### Handheld dynamometer hardware and software

A handheld dynamometer (HHD) recording at 40 Hz was used to capture force output data (Lafayette Manual Muscle Tester, Lafayette Instrument Co. Sagamore, IN). Data was extracted from onboard storage and converted for analysis using the manufacturer’s MMTDownload Tool on Microsoft Windows software. The dynamometer transducer was set at a 1-pound activation threshold and a zero-pound deactivation threshold.

### Testing protocol

#### Sensor application

The skin was prepped per standard protocol for electromyography with an alcohol wipe. Surface EMG sensor placement included the medial head of the gastrocnemius and the rectus femoris, and for the hamstrings, we selected either the biceps femoris or the semitendinosus muscle based on which elicited the larger muscle bulk contraction during resisted knee flexion (most frequently the biceps femoris). The sEMG devices were applied to the skin following the Delsys manufacturer application guidelines, and standardized sEMG placement locations were used for each muscle group (De Luca 2002). The sEMG sensors were applied using a medical grade, clear, double-coated adhesive polyethylene.

**Figure 2.**
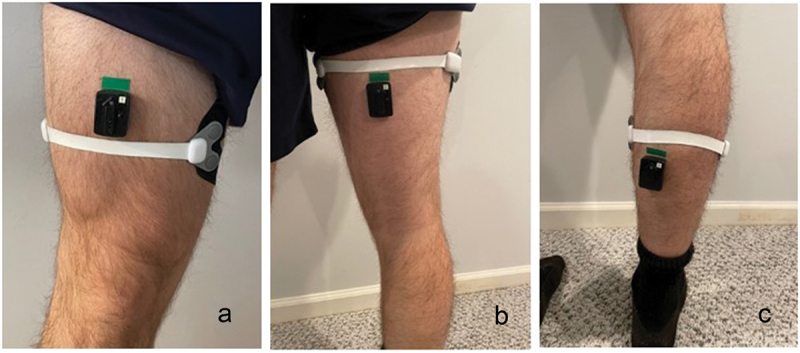
EMG and sMMG sensor applications for the quadriceps (a), hamstrings (b), and gastrocnemius (c).

The sMMG sensors were applied with athletic tape (medical grade adhesive) and were placed perpendicularly across the most prominent portion of the muscle bulk. A minimal amount of tension was applied to the elastic component of each sMMG sensor without overstretching it. To standardize the placement of the quadriceps sensor, the subjects were placed in a seated position with their right leg out straight. The subjects were asked to place their left hand across their body over the top of their kneecap, such that their 5th digit contacted their kneecap, while their thumb was placed proximally a few inches up their thigh. The sMMG sensor was then placed along the side of the subjects’ thumb, extending laterally across the bulk of the quadriceps musculature (Figure 2(a)). The hamstring sensor was placed by asking the subjects to stand holding onto the back of a chair for support while lifting one foot from the ground to bend their knee. The sMMG sensor was placed across the largest area of the hamstring muscle bulk (Figure 2(b)). The gastrocnemius sMMG was placed by having the subjects rise up on their toes. The sensor was placed across the largest bulk of both heads of the gastrocnemius (Figure 2(c)).

### Test protocol

**Figure 3.**
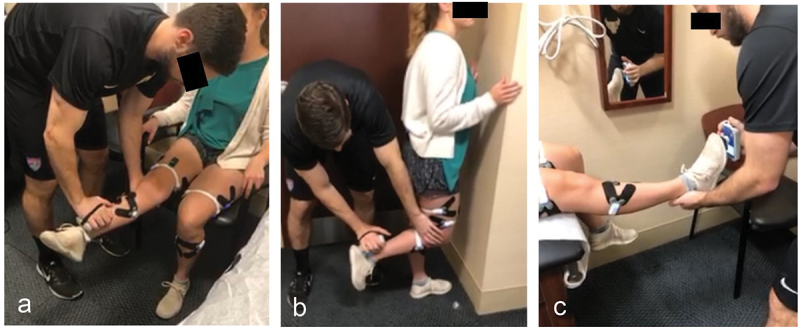
Testing positions for the isometric contraction testing. Handheld dynamometry positioning is shown for the quadriceps (a), hamstrings (b) and the gastrocnemius (c).

Subjects performed a series of maximal volitional isometric contractions (MVIC) during unilateral resisted trials for each muscle group. Each subject was asked to perform five trials for each muscle tested. The HHD was applied following standardized muscle testing ‘break test’ protocols where maximum resistance force was applied across the distal portion of the resisted body segment (Andrews et al. 1996), and the force generated in the opposing direction of the applied force was recorded. For each muscle, the subject was asked to ‘push against the dynamometer as strongly as possible at a “go” verbal command and continue pressing until told to relax or the tester’s force overcomes the subject’s force production’, which occurred after approximately 3 s (Figure 3).

#### Quadriceps force testing protocol

The subject was asked to sit upright with ¾ of their thighs supported on a chair. Each subject was asked to straighten the limb being tested so that it was extended in the air, while the opposite limb was positioned with approximately 90° knee flexion with their foot planted on the ground (Figure 3(a)) (Kendal FP & McCreary EK, FP and McCready 1983). The dynamometer transducer was placed approximately 3 cm above the ankle joint line (Andrews et al. 1996).

#### Hamstrings force testing protocol

The subject was asked to stand with their hands against a wall for stability. Their left leg would remain planted firmly on the ground, while their right leg would raise up into 90 knee flexion during the test. The dynamometer was placed approximately 3 cm above the ankle joint line on the posterior side of the limb.

#### Gastrocnemius force testing protocol

The subject was asked to lie supine on a plinth to allow their entire body to be supported except for the lower shank. The transducer was placed on the sole of the foot (proximal to the metatarsal heads) (Spink et al. 2010).

#### Bilateral squat activity

**Figure 4.**
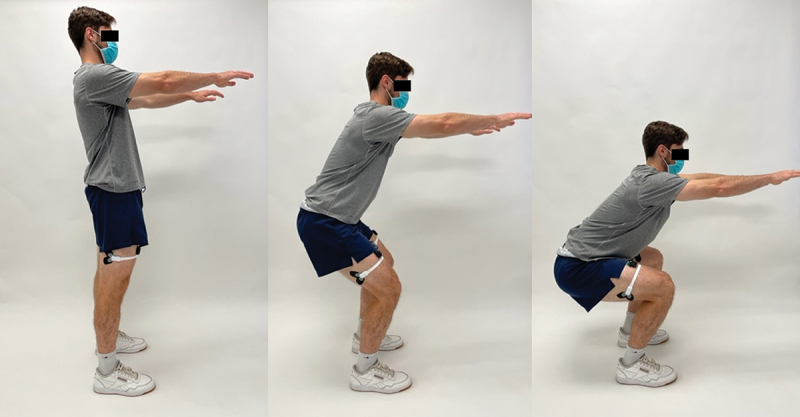
Illustration of the parallel squat activity.

After each participant completed the maximum isometric contraction tests, they were asked to perform a set of three parallel squats. The activity started with each participant standing with their feet positioned shoulder width apart. Prior to the start of motion, each person was asked to put their arms out straight in front of them at shoulder height (Figure 4). Participants were requested to lower themselves into a squat at their natural pace and comfortable level of depth without allowing their heels to lose contact with the ground. Subjects were asked to squat as low as possible to a range where their thighs would be parallel to the ground and prevent their posterior thigh and calves to touch (preventing sensor contact). Each participant completed at least three squats for a total of 45 trials across the 15 participants.

### Data analysis

**Figure 5.**
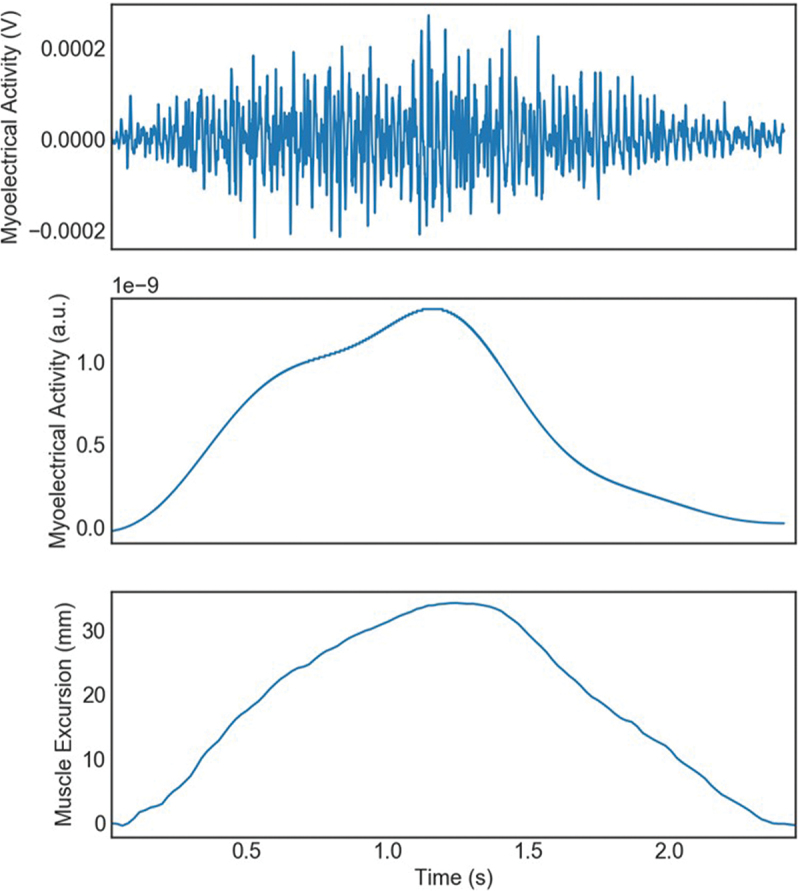
Raw quadriceps contraction data output from a parallel squat sEMG (top), filtered sEMG (middle) used for data analysis comparison to raw sMMG (bottom).

**Figure 6.**
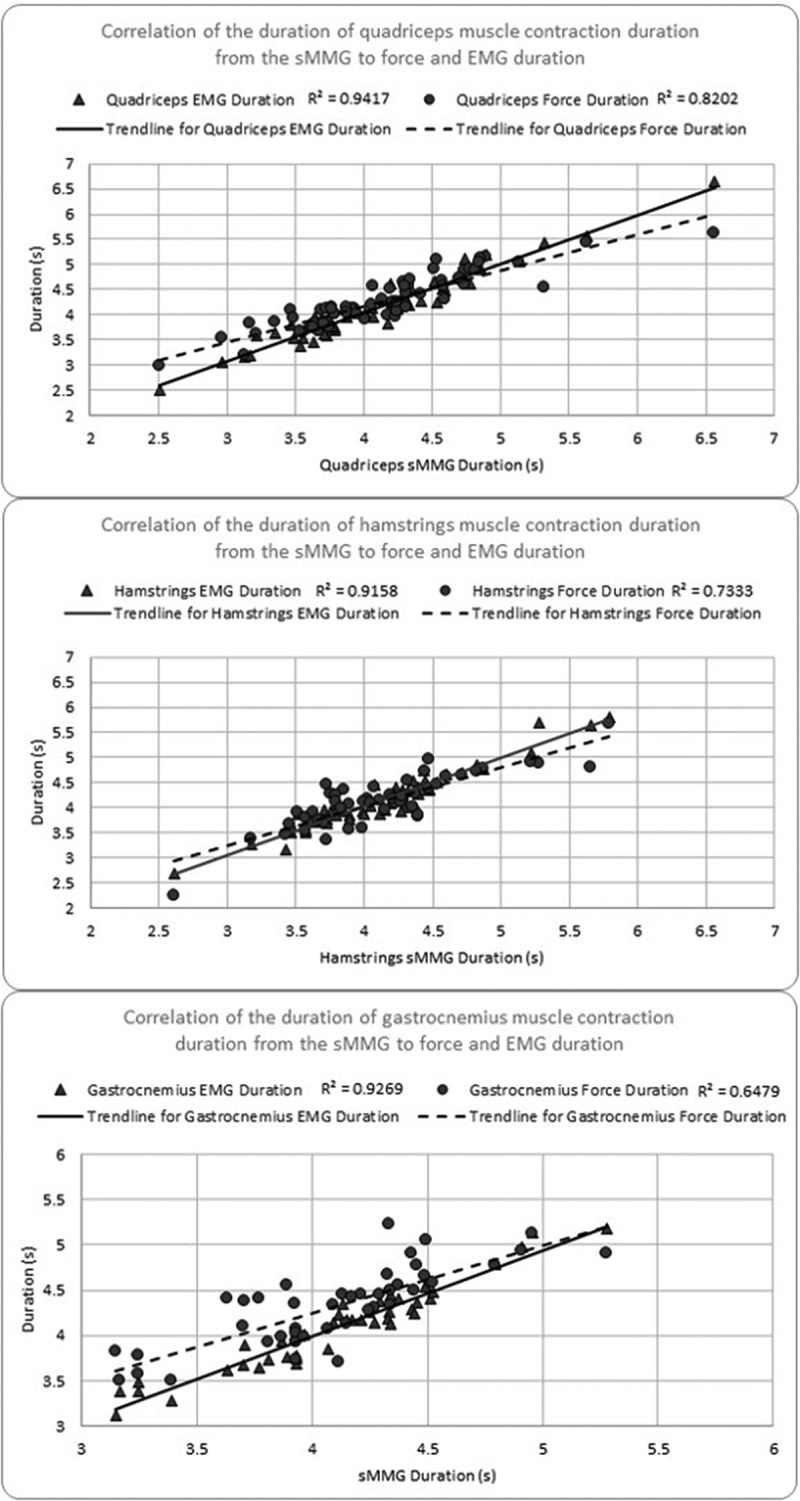
Correlation plots of sMMG contraction duration compared to force HHD (dotted line) and EMG (solid line) for quadriceps (a), hamstrings (b) and gastrocnemius (c).

For a participant’s data to be included in the analyses, a minimum of three trials were required to demonstrate successful data capture from all three measurement methods, per muscle group. Trials were omitted due to low subject effort, inconsistency with maximal contractions and artifact in the EMG signal. Some subjects refused performing five isometric trials due to fatigue. Our isometric muscle contraction data collection resulted in a total of 161 trials total across all subjects and all muscles (Table 1). There was a total of 54 trials for parallel squat quadriceps data captured for data analyses (Figure 5). Raw sMMG and dynamometer data were used in all analyses, while signal processing was applied to sEMG data (Figure 6). A rectified Teager-Kaiser Energy Operator was first applied to sEMG data to accurately determine the onset of electrical activation. This operator aggregates local energy and completes the full wave rectification of the signal, making the detection of timing events easier by improving the signal-to-noise ratio (Solnik et al. 2008; Boudraa and Salzenstein 2018). The Teager-Kaiser Energy Operator has been reported effective in the literature for EMG signals and has been shown to improve the accuracy of threshold-based detection of the onset of EMG signals (Solnik et al. 2008; Boudraa and Salzenstein 2018). The sEMG data was then processed with a low-pass sixth-order Butterworth filter at 3 Hz prior to timing analyses to remove motion artifacts and provide a smoothed envelope of the original signal.

**Table 1.** Results of Pearson R correlation coefficient analyses of the average muscle activation duration (all trials *n* = 161) for the quadriceps, hamstrings and gastrocnemius muscles, across the three measurement methods for 15 persons.

Muscle	sMMG signal duration (s)	sEMG signal duration (s)	Force signal duration (s)
Quadriceps* n = 63 trials	4.14 ± 0.66	4.17 ± 0.66	4.27 ± 0.52
Hamstrings* n = 52 trials	4.14 ± 0.59	4.16 ± 0.59	4.13 ± 0.54
Gastrocnemius* n = 46 trials *	4.17 ± 0.61	4.16 ± 0.63	4.38 ± 0.49

*Indicates the Pearson R correlation coefficient was statistically significant at *p* < 0.05.

The time point of muscle activation and deactivation was set at a threshold value three times the standard deviation of the standing calibration trial above the minimum value for sMMG and sEMG. The handheld dynamometer’s activation threshold was set at one pound, such that it was low enough to detect the initial onset of force, but high enough to avoid detection of any slight contact that may occur prior to true activation. The timepoints of muscle activation and deactivation were calculated using all three modalities. Since contraction duration was the desired metric, the three sensor systems did not need to be synchronized as duration is independent from time synchronization.

ANOVA statistical analyses compared total duration of contraction time measured by sMMG and sEMG, respectively, for each subject’s quadriceps, hamstrings, and gastrocnemius muscle bulks during the parallel squat and MVIC activities. Total duration of contraction was calculated as the duration between the timepoints of muscle activation and deactivation as defined above that were independently measured by sMMG and sEMG. Pearson correlation coefficient analyses were performed to identify the relationships between the contraction duration times from sMMG, sEMG and dynamometry force output. The threshold of statistical significance was established at *p* < 0.05.

## Results

### Correlation of the duration time of musculature activation between sMMG, sEMG and force dynamometry

**Figure 7.**
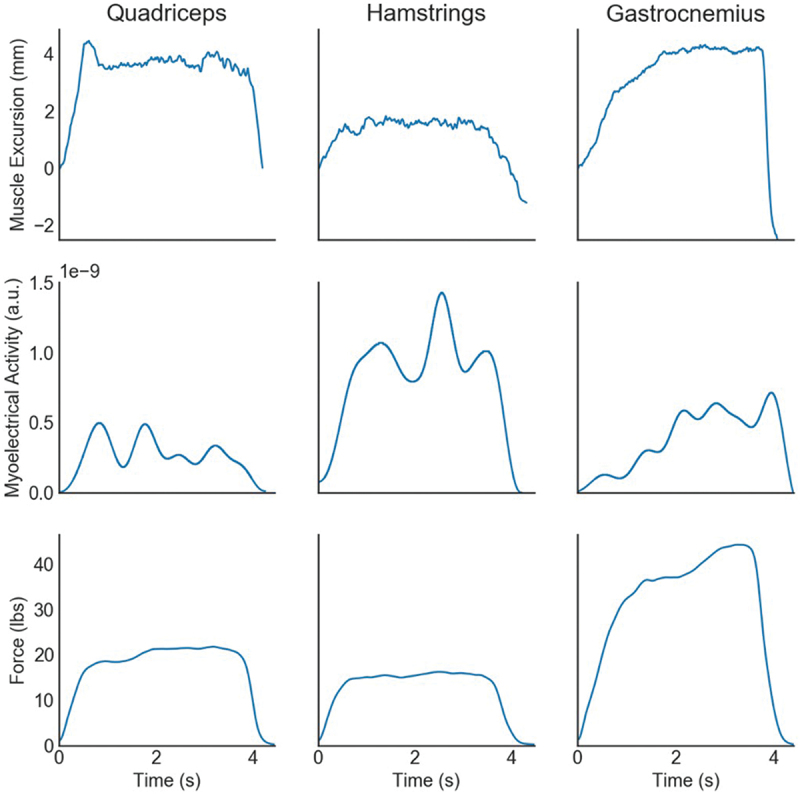
Sample data for illustration comparing contraction output across the three methods of capturing contraction duration: sMMG (top row), filtered EMG (middle row), and force output (bottom row) for the quadriceps (left), hamstrings (middle), and gastrocnemius (right).

The results of the sMMG signal duration when compared to the sEMG signal duration during a MVIC of the quadriceps musculature demonstrated a strong correlation *r* = 0.970, *p* < 0.001 (Table 1, Figures 5 and 6). Similarly, the sMMG signal duration showed a strong correlation with the handheld dynamometer force signal duration output, *r* = 0.906, *p* < 0.001. The raw data signals for all three measurement devices align well as seen in Figure 7. During the MVIC of the hamstring musculature, the duration of the sMMG signal revealed strong correlation with the sEMG signal duration, *r* = 0.958, *p* < 0.001 (Table 1 and Figure 6). The duration of the sMMG signal when compared to the force output signal duration from the handheld dynamometer during a MVIC of the hamstrings musculature demonstrated a strong correlation *r* = 0.856, *p* < 0.001. The duration of the sMMG signal compared to the sEMG signal duration during a MVIC of the gastrocnemius musculature also demonstrated a strong correlation *r* = 0.979, *p* < 0.001 (Table 1 and Figure 6). During the MVIC of the gastrocnemius, the duration of the sMMG signal revealed moderate correlation with the output signal duration from the handheld dynamometer, *r* = 0.728, *p* < 0.001.

### Correlation of the duration time of musculature activation between sMMG and EMG during the parallel squat

The data produced by both the sMMG and the sEMG yield similarly displayed data output patterns (Figure 5). There was a strong correlation of the total duration of quadriceps contraction as detected by the sEMG (mean = 2.526 ± .519 s) and sMMG (mean = 2.489 ± 0.504 s) during the parallel squat, *r* = 0.822, *p* < 0.001.

## Discussion

Our study findings revealed that the duration of a muscle contraction captured by the novel sMMG sensor demonstrated a strong correlation with the duration time collected from the sEMG across all three muscle groups during the MVIC testing, as well as with the quadriceps muscle during the parallel squat activity, supporting our first hypothesis. Similarly, our second hypothesis was supported as a moderate correlation was established between the duration of muscle contraction from the sMMG and the duration time captured by the force output from the dynamometer. Evaluation of isometric activities was chosen for this study to maximize standardization of subjects’ movements and related pattern of muscle activation while minimizing possible motion artifacts from dynamic motions that have been shown to confound prior sEMG analyses (Nazmi et al. 2016). We successfully demonstrated moderate correlation of quadriceps contraction duration timing between sMMG and sEMG during a relatively slow and common movement evaluation activity, the parallel squat. This finding suggests good potential for the sMMG to be used in more active clinical applications. The successful simultaneous recording from the sMMG and dynamometer force output encourages future exploration of the magnitude of muscle contraction excursion and force production during exercise and functional activities in clinical application.

Study results suggest the ability of sMMG sensors to both complement and improve upon the use of current sEMG analysis. Evaluation of muscle contraction timing was carried out using the raw sMMG data without any of the complex signal processing required of sEMG analysis. The nature of the sMMG mechanical signal may also help to avoid some well-established data collection challenges of sEMG including the need for minimization of potential sources of signal interference and processing required for the electrical sEMG signal (Madeleine et al. 2001). Application of sEMG in rehabilitation settings is hampered by such challenges (Pilkar et al. 2020). The form fitting aspect of the sMMG may have limited artifact during the presented study data recording.

The novel sMMG sensor may offer benefits over other wearable sensors that aim to evaluate muscle function via measurements of physical muscle displacement. In the past, this has been accomplished using technology such as piezoresistive sensors and muscle circumference sensors (Kim et al. 2014; Esposito et al. 2018). One potential benefit is that the sMMG is secured with athletic tape making the application of the sensor easy and secure. In our study, the sensors stayed secure throughout the transitions from sitting and standing positions during the testing session as well as during the parallel squat activity trials. Most MMG sensors have difficulty conforming to body segments which may make application for clinical use during activities of standing and dynamic motion more challenging and less comfortable (Bar-Cohen 2002).

Current MMG sensors composed of force sensitive resistors applied superficially to the skin have been used to detect the force produced by underlying contracting muscles (Bar-Cohen 2002; Esposito et al. 2018). Investigations of the force-sensitive resistors report mechanical creep during loading which results in output drift error (Paredes-Madrid et al. 2017; Esposito et al. 2018). Such MMG systems are likely best utilized for short data collection times similar to those performed during the 3 s isometric trials in our current study. Previous publications using the dielectric elastomer (like the sMMG sensors used in our study) for use in other applications have not reported difficulty with creep (Bar-Cohen 2002; Wang et al. 2016). Future studies are warranted to assess creep during application of the newly introduced sMMG during dynamic activities compared to other MMG devices. The new sMMG sensor may have fewer limitations than those previously described for a muscle circumference MMG sensor when applied to the lower limb. The muscle circumference MMG sensor is described for use on the upper limb, which due to the small deeper muscles, may be better suited than the current version of the sMMG sensor. The muscle circumference sensor is composed of elastomers that measure muscle contraction by assessing changes in muscle cross-sectional area (Kim et al. 2014). It is currently used for human robot interfaces, harnessing the rapid signals captured around the total surface of a limb prior to intentional movement to elicit movement of prosthetic devices (Kim et al. 2014). Calculations using the circumference sensor are dependent on a process that converts measured changes in muscle circumference into muscle activity data focused on the upper extremity (Kim et al. 2014). Since the sMMG sensor overlays only the muscle bulk of interest and records changes in muscle bulk displacement as opposed to the limb girth circumference, sMMG use for forearm muscles, which overlay each other, will require separate validation.

In addition to the timing duration of muscle activation, from the sMMG sensor, the measure of muscle output (i.e. total muscle bulk excursion) with force generation may also have applications for evaluating changes in muscle contractility during exercise and functional activity. Other mechanomyography methods such as tensiomyography have already demonstrated how the magnitude of radial muscle displacement can reveal changes in muscle contractility behavior following strength training (de Paula Simola Rà et al. 2015).

The correlation of our sMMG sensor output with simultaneous force output is also consistent with prior literature findings where other mechanomyography signal types have demonstrated good visual association of peak force production and timing of activation and relaxation during MVICs (Cé et al. 2015). The sMMG may be helpful in the identification of muscle overexertion as associated with muscle fatigue. Currently, combined sEMG and force production analysis is one of the most common methodologies for assessing muscular exertion (Scherrer and Bourguignon 1959; Cè et al. 2020). The sMMG sensor analysis displays timing patterns consistent with sEMG signals, suggesting that sMMG sensors may have use in the assessment of peripheral muscle overexertion. In this case, peripheral or superficial muscular overexertion refers to the reduced ability of skeletal muscle to generate force with the same work demands (Place et al. 2010; Cè et al. 2020). There is also potential application of the sMMG sensor for monitoring muscle recovery after injury. Future exploration grading the sMMG output may lead to insights on muscle contraction fluctuation associated with overexertion.

Extrapolation of the results of this study is limited to the muscles assessed during the MVICs and the parallel squat in a controlled environment. Analysis of isometric contractions was chosen in this study to evaluate timing correlations of simultaneous output measures across three measurement modalities. Using a HHD to assess powerful muscle groups can be a challenge for the tester, especially for the gastrocnemius muscles. While we are confident in consistent stabilization during our testing, it is plausible that the muscle contractions were not exerted to maximum potential. For the purpose of capturing simultaneous muscle activation time output across the three measurement tools, we are confident in the methodology used. The magnitude of sMMG signal output was not evaluated in comparison to the magnitude of force output or magnitude in sEMG output in this initial study of isometric muscle contractions. The sMMG sensor investigated in our study captured data at 50 Hz which provided our study with good results for the isometric and parallel squat activity. However, this is a relatively low sampling rate to capture muscle activation for quicker paced activities or exploration of muscle pathology. Future exploration of the sMMG with faster capture rates will be advantageous to investigate progressive applications of this technology.

This feasibility study has a modest sample size and presents the content validity of the sMMG sensor output. The study establishes the potential application of the sMMG sensor for capturing the duration timing of a muscle contraction, like sEMG. However, the sMMG presents with the added benefits of easy and form fitting sensor application and no need for complex signal processing. The sMMG, which was easily donned, may facilitate data capture of timing of lower extremity muscle contractions for use in monitoring recovery during rehabilitation. Successful sMMG detection of muscle contraction is supported by similarity to sEMG and dynamometer output, demonstrating content validity during isometric muscle contractions.

## Data Availability

Due to the nature of this research, participants of this study did not agree for their data to be shared publicly, so supporting data is not available.
